# Protein Interactions Network of Hepatitis E Virus RNA and Polymerase With Host Proteins

**DOI:** 10.3389/fmicb.2019.02501

**Published:** 2019-11-01

**Authors:** Gayatri D. Kanade, Kunal D. Pingale, Yogesh A. Karpe

**Affiliations:** ^1^Nanobioscience Group, Agharkar Research Institute, Pune, India; ^2^Savitribai Phule Pune University, Pune, India

**Keywords:** host-protein interactions, protein interactions network, system biology, gene ontology analysis, hepatitis E virus, viral RNA, viral polymerase

## Abstract

Host-pathogen interactions are crucial for the successful propagation of pathogens inside the host cell. Knowledge of interactions between host proteins and viral proteins or viral RNA may provide clues for developing novel antiviral strategies. Hepatitis E virus (HEV), a water-borne pathogen that causes acute hepatitis in humans, is responsible for epidemics in developing countries. HEV pathology and molecular biology have been poorly explored due to the lack of efficient culture systems. A contemporary approach, to better understand the viral infection cycle at the molecular level, is the use of system biology tools depicting virus-host interactions. To determine the host proteins which participate in the regulation of HEV replication, we indentified liver cell proteins interacting with HEV RNA at its putative promoter region and those interacting with HEV polymerase (RdRp) protein. We employed affinity chromatography followed by liquid chromatography quadrupole time-of-flight mass spectrometry (LC-QTOF/MS) to identify the interacting host proteins. Protein-protein interaction networks (PPI) were plotted and analyzed using web-based tools. Topological analysis of the network revealed that the constructed network is potentially significant and relevant for viral replication. Gene ontology and pathway enrichment analysis revealed that HEV RNA promoter- and polymerase-interacting host proteins belong to different cellular pathways such as RNA splicing, RNA metabolism, protein processing in endoplasmic reticulum, unfolded protein response, innate immune pathways, secretory vesicle pathway, and glucose metabolism. We showed that hnRNPK and hnRNPA2B1 interact with both HEV putative promoters and HEV RdRp, which suggest that they may have crucial roles in HEV replication. We demonstrated *in vitro* binding of hnRNPK and hnRNPA2B1 proteins with the HEV targets in the study, assuring the authenticity of the interactions obtained through mass spectrometry. Thus, our study highlights the ability of viruses, such as HEV, to maneuver host systems to create favorable cellular environments for virus propagation. Studying the host-virus interactions can facilitate the identification of antiviral therapeutic strategies and novel targets.

## Introduction

Hepatitis E virus (HEV) is a hepevirus that is transmitted via contaminated drinking water to cause acute hepatitis in humans. Although prevalence of HEV has been mainly observed in developing countries, its spread has been reported in many industrialized countries across the globe in recent years. In infected adults, mortality rate due to HEV is up to ∼2% while in infected pregnant women, it increases up to 30% (Meng, 2010; Nan and Zhang, 2016). Due to the lack of efficient culture systems and robust animal models for HEV propagation, molecular mechanisms underlying the HEV lifecycle are not known (Himmelsbach et al., 2018). Non-specific treatment with pegylated interferons along with ribavirin is recommended in rare instances for severe cases because specific antiviral drugs or vaccines against HEV are still not available worldwide (van de Garde et al., 2017).

Hepatitis E virus belongs to the Orthohepevirus genus, and its genome consists of positive sense, single-stranded RNA comprised of three open reading frames (ORFs) (Kenney and Meng, 2018). First step of HEV replication cycle is the translation of ORF1 present on the positive sense genomic RNA to form non-structural polyprotein, which consists of functional domains of methyltransferase, protease, helicase and RNA-dependent RNA polymerase (RdRp). Two additional ORFs present in the sub-genomic intermediate RNA, ORF2 and ORF3, encode capsid protein and a small multifunctional phosphoprotein, respectively (Kenney and Meng, 2018). HEV replication cycle involves the formation of a negative sense RNA complementary to the positive sense genomic RNA. HEV RdRp recognizes and starts the transcription at the promoter present at the 3′ end of the positive sense strand to form a complementary negative strand RNA. Negative strand RNA bears two putative promoters: one is the genomic promoter (3′ end of negative sense RNA) for the synthesis of positive sense RNA and the other is the sub-genomic promoter for the synthesis of sub-genomic RNA. Transcription at different viral promoters has to be regulated to maintain the correct stoichiometry of positive sense, negative sense and sub-genomic intermediate RNA. Host proteins, binding at viral promoters as components of the viral replicase complex, help in the regulation of molecular switches responsible for maintaining viral RNA stoichiometry, and their temporal synthesis.

Viruses bear relatively compact genomes, encoding a limited number of proteins and, therefore, rely on host factors to establish replication in the infected cell. Being obligatory intracellular parasites, viruses have to subvert the biosynthetic pathways of the host cell. Constant interactions between the virus and its host during the process of co-evolution have shaped the anti-viral immune system of the host and, in turn, the capability of viruses to manipulate host control mechanisms to facilitate their propagation (Stebbing and Gazzard, 2003; Fermin and Tennant, 2018). Classical scientific approaches to understanding the molecular basis of such virus-host interactions involve analysis of individual gene or protein targets and study of their functional significance. However, these approaches have not been sufficient to address the challenges of the host-pathogen interface. System biology tools provide a multidimensional approach for a comprehensive view of the biological system at molecular network levels. High throughput genomic and proteomic studies, such as siRNA and microRNA screens, and microarrays have greatly expanded our understanding of virus-host networks. Advances in several tools for data acquisition, processing, integration and computation provide rapid, and promising strategies for the development of new therapies for infectious diseases (Peng et al., 2009; Aderem et al., 2011; Xue and Miller-Jensen, 2012).

Studies on several viruses reveal that viral proteins or viral RNA interact with host proteins to regulate viral replication. Previous studies on the HEV ORF1 and ORF2 interactome showed the involvement of factors associated with different biological processes, such as ubiquitin proteasome system, innate immunity and RNA metabolism (Ojha and Lole, 2016; Subramani et al., 2018). In another study by Paingankar et al. host factors have been found to interact with the untranslated region on HEV genomic RNA (Paingankar and Arankalle, 2015). However, no conclusive studies have been carried out to analyze host factors present in HEV replicase complex. Also, host factors interacting with promoter sequences on HEV negative sense RNA have not been explored so far. We hypothesized that the set of host proteins interacting with HEV polymerase protein and HEV RNA at its promoter region must play crucial roles in tightly regulating the synthesis of viral RNAs. The host factors may form differential replicase complexes along with HEV RNA and polymerase protein at the promoter region. Therefore it was interesting to find out the proteins which bind at both the genomic and sub-genomic promoters on negative sense RNA of HEV and those which bind to only one specific promoter along with the RdRp. Proteins binding at both the promoters may act as primary transcription factors while, the differential proteins may guide RdRp for where to bind and which strand to synthesize at a given time. We thus believe that host factors interacting with HEV polymerase and promoters play crucial roles in regulating the molecular switches in HEV replication.

In order to better understand the HEV-host interface, we identified liver cell proteins interacting with the HEV polymerase and HEV putative promoters and generated a protein-protein interaction network. We further utilized a bioinformatics approach to analyze these interaction networks and assess their significance. Our study identified host proteins related to cellular processes like RNA metabolism, unfolded protein response, stress granules, secretory vesicles, endoplasmic reticulum protein processing, and innate immune pathways. HNRNPK and HNRNPA2B1 proteins were found to be interacting with both HEV promoters and HEV RdRp. We demonstrated the *in vitro* binding of HEV promoters and HEV RdRp with HNRNPK and HNRNPA2B1, confirming the validity of interactions obtained by mass spectrometry.

## Materials and Methods

### Virus Replicon and Cells

Infectious replicon of Sar55 strain of genotype 1 of HEV (pSK-HEV2) and a subclone of a human hepatoma cell line Huh7 S10-3 which is permissive for the replication of HEV infectious clone was obtained from Dr. Suzanne U. Emerson, NIH, Bethesda, MD, United States. Cells were maintained in DMEM GlutaMAX (Invitrogen) medium supplemented with 10% fetal bovine serum (FBS) (Invitrogen) and 100 U/ml penicillin and 100 mg/ml streptomycin (Sigma).

### Construction of Recombinant Plasmids

Coding sequence of HEV RNA dependent RNA polymerase (RdRp) was amplified from pSK-HEV2 replicon. RdRp coding sequence was cloned in pcDNA 3.1/myc-His (-) mammalian expression vector in such a way that it will be expressed as FLAG tagged RdRp at its N terminal. This clone has been designated as pcDNA_FLAG-RdRp. Primers used for the amplification have been listed in Table 1.

**TABLE 1:** List of primers used in the study.

**Sr. No.**	**Primer name**	**Sequence (5′-3′)**	**Amplified construct**
1	pTandem-F	GCTAGCCAAGCGCTTGGTTAAC	pTandem
2	pTandem-R	CCATGGTGGCATATCTCC	
3	RdRpFLAG_pTF	TTAAGAAGGAGATATGCCACCATGGCGCCACCATGGACTACAAAG	RdRp
4	RdRpFLAG_pTR	TGGTGATGGTGTGTCATTCCACCCGACACAG	
5	RdRpFLAG_ pTJncF	TCGGGTGGAATGACACACCATCACCACCATC	Junction region
6	RdRpFLAG_ pTJncR	TTTGTTCCATGTTGTTTAAACTTTCAAAGGAAAACCAC	
7	HNRNPK_F	TTTCCTTTGAAAGTTTAAACATGGAAACTGAACAGCCAG	hnRNPK
8	HNRNPK_R	TAACCAAGCGCTTGGCTAGCTTACAGATCCTCTTCTGAGATG	
9	HNRNPA2B1_F	TTTCCTTTGAAAGTTTAAACATGGAGAGAGAAAAGGAACAGTTC	hnRNPA2B1
10	HNRNPA2B1_R	TCTGAGATGAGTTTTTGTTCGTATCGGCTCCTCCCACC	
11	RdRp_F	ATCCGAATTCATGGACTACAAAGACGATGACGACAAGGGTGGCGAAATTGGCCACCA	pcDNA_FLAG-RdRp
12	RdRp_R	CGGAGGGATCCTCATTCCACCCGACACAGAATTGA	
13	G promoter_F	GCAGACCACATATGTGGTCGATGCCATGGA	G promoter
14	G promoter_R	GACTACTAATACGACTCACTATAGGGAGAAAAGGCCTAACTACC	
15	Sg promoter_F	AGTCAGTGAAGCCAGTGCTTGACCTGACAAATTCAATTCTGTGT	Sg promoter
16	Sg promoter_R	GACTACTAATACGACTCACTATAGGGCGGGCAGCATAGGCAGAA	

To confirm interaction of HEV RdRp with hnRNPK/hnRNPA2B1, FLAG tagged RdRp and c-Myc tagged hnRNPK or hnRNPA2B1 encoding sequence was cloned in pTandem vector (Clontech) under CMV promoter and IRES, respectively. The constructs have been designated as pTandem_FLAG-RdRp_Myc-hnRNPK or pTandem_FLAG-RdRp_Myc-hnRNPA2B1. Primers used for the cloning are mentioned in the Table 1.

### Preparation of HEV Promoter RNA Baits

Sequences coding for the putative genomic promoter (G promoter: nt 1 to 139 on positive sense RNA) and putative sub-genomic promoter (Sg promoter: nt 5051 to 5200 on positive sense RNA) of HEV genotype 1 were PCR amplified from pSK-HEV2 replicon. The primers for the amplification of template were designed with T7 promoter sequence in such a way that the RNA of anti-sense orientation is generated. Primers used for the amplification have been listed in Table 1. PCR products were used as templates for the synthesis of RNAs bearing respective promoter sequences. *In vitro* RNA was synthesized by using MEGAscript kit (Ambion) following the manufacturer’s instructions. Biotinylated *in vitro* transcribed RNAs were prepared using 5 mM rATP, 5 mM rGTP, 5 mM rUTP, 4.5 mM rCTP, and 0.5 mM of biotin-14 CTP (Invitrogen) in the rNTP mix for the *in vitro* transcription reaction. For synthesizing non-biotinylated RNAs of respective regions, total 5 mM rCTP was added instead of biotin-14-CTP. Unincorporated nucleotides were removed by purifying the RNA using phenol-chloroform precipitation method. Purified RNAs were visualized on 2% agarose gel.

### RNA Affinity Chromatography

A total of 2 μg of each of biotinylated RNA corresponding to either HEV putative genomic or sub-genomic promoter were coupled with M280 streptavidin dynabeads (Invitrogen) in the presence of nucleic acid binding and washing buffer (B&W buffer: 10 mM Tris–HCl, pH 7.5, 1 mM EDTA, 2M NaCl) for 15 min at room temperature on a rotator. Before RNA binding step, beads were washed with solution A (DEPC-treated 0.1 M NaOH, 0.05M NaCl) followed by solution B (DEPC treated 0.1 M NaCl) to remove RNase. Huh7 S10-3 cells were harvested at ∼80% confluency in the lysis buffer (10 mM Tris–Cl, pH 7.4, 10 mM KCl, 2 mM MgCl_2_, 0.5% Tritin X-100 with protease inhibitor cocktail). The lysate was prepared by centrifugation at 12000 rpm at 4°C for 20 min. The bound RNA-beads complexes were incubated with Huh7 S10-3 cell lysate pre-cleared with 20 μl beads for 1 h at 4°C. Cell lysate and RNA-beads complexes were mixed and incubated together at 4°C on a rotator for 2 h. Bound complexes were washed with B&W buffer and proteins bound to RNA were eluted in 100 μl elution buffer (50 mM Tris–Cl, pH 7.4, 0.2% SDS, 0.1% Tween 20). Eluted proteins were loaded on 12% SDS PAGE followed by silver staining for visualization of protein bands using ProteoSilver staining kit (Sigma). Eluates from three independent RNA affinity chromatography experiments were pooled together and subjected to protein identification by mass spectrometry.

### Immunoprecipitation

pcDNA_FLAG-RdRp construct was transfected into Huh7 S10-3 cells using Lipofectamine 3000 (Invitrogen) transfection reagent. After 48 h post transfection, cells were harvested and lysed in IP lysis buffer (50 mM Tris–Cl pH 7.4, 150 mM NaCl, 1% IGEPAL and protease inhibitor cocktail). Protein G dynabeads (30 μl; Invitrogen) were used for each immunoprecipitation experiment. Next, 4 μg of either rabbit anti FLAG antibody or a non-specific isotype IgG was incubated with washed dynabeads for 30 min at room temperature on a rotator. The cell lysate was incubated with antibody plus dynabeads complex for 2 h at 4°C on a rotator. Three washes with IP lysis buffer were given, followed by a final wash with PBS. Interacting proteins were eluted in 50 μl of elution buffer (50 Mm Tris–Cl pH 7.4, 0.2% SDS, and 0.1% Tween 20). Eluted proteins were loaded on 12% SDS PAGE followed by silver staining for visualization of protein bands using ProteoSilver staining kit (Sigma). Eluates from three independent immunoprecipitation experiments were pooled together and subjected to protein identification by mass spectrometry.

### Liquid Chromatography Quadrupole Time-of-Flight Mass Spectrometry (LC-QTOF/MS)

Hepatitis E virus RNA-dependent RNA polymerase and RNA interacting proteins isolated by immunoprecipitation and RNA affinity chromatography, respectively, were subjected to liquid chromatography-mass spectrometry (LC-MS) analysis. Total 30 μg of each of the protein samples was acetone precipitated, and the protein pellets were dissolved by adding 10 μl of 8 M urea, and the volume was brought to 15 μl with water. Samples were then reduced by the addition of 1.5 μl of 100 mM DTT and heated at 90°C for 15 min. The sample was cooled and alkylated by adding 1.5 μl of 200 mM IAA and incubated in the dark at RT for 15 min. 82 μl of ABC was added, and proteins were digested by adding 1 μl of 1 mg/ml trypsin protease and incubating at 37°C for 16 h. The reaction was stopped by the addition of 1–2 μl of concentrated TFA. Then peptides were dissolved in 0.1% TFA, 5% ACN in water for MS-analysis. Agilent 1260 infinity HPLC-Chip/MS system is a microfluidic chip-based technology was used for peptide enrichment and separation. Charged peptides from HPLC-Chip system were directly infused into mass-spectrometer for detection. Agilent Mass Hunter software was used for data acquisition and analysis of total ion chromatograms. Protein searches were carried out using Morpheus software. Protein identification was performed with the following criteria: (a) Trypsin digested peptides with 2 missed cleavages allowed, (b) peptide tolerance <10 ppm, (c) >2 unique peptides, (d) FDR <1%. Fasta files for Human Proteome database were downloaded from the UniProt was used for protein searches. Proteins found in respective negative control sample were eliminated from the dataset to remove non-specific interactions.

### Construction of the Molecular Interaction Network

All the experimentally derived data sets were used to generate HEV-host proteins interaction network by using “Cytoscape version 3.6.1” (Shannon et al., 2003). To analyze the interaction among host proteins, IntAct protein interaction database was used. Only interactions confirmed by direct physical binding were considered for plotting inter protein interaction map. Topological parameters and central measures of the network were calculated by using a network analyzer tool in Cytoscape. Human protein-protein interaction analysis was also performed by using STRING database. In all the networks and throughout the study, we have used NCBI gene names to represent the proteins in order to have a consensus in protein accession. Corresponding gene names, protein names, and Uniprot protein identifiers have been listed separately (Supplementary Tables 1a–c).

### Gene Ontology Analysis

Gene ontology “GO” analysis was carried out by using web-based tools like Panther (Gene Ontology Consortium’s web tool), Gprofiler, STRING, and Enricher. To analyze the enrichment of specific pathway, KEGG annotation database was used. Gene set and pathway enrichment analysis was validated by Fisher’s exact *t*-test. To control false discovery rate (FDR) Benjamini and Hochberg multiple test correction was used; *p*-value ≤ 0.05 was considered significant. The corrected *p*-value for each GO term has been given in the section “Results.”

### Co-immunoprecipitation for Validation of Interactions *in vitro*

Huh7 S10-3 cells expressing FLAG-RdRp and Myc-hnRNPK/hnRNPA2B1 were harvested at 48 h post transfection and resuspended in IP lysis buffer (50 mM Tris–Cl, pH 7.5, 150 mM NaCl and 1% IGEPAL detergent) for half an hour at 4°C. Cell lysate was prepared by centrifugation at 12000 rpm for 30 min. Clear supernatant was mixed with anti Myc antibody or isotype IgG antibody attached to protein G dynabeads (Invitrogen) for 2 h. Three washes of IP lysis buffer were given to remove non-specific interactions. Interacting RdRp-host cellular protein complexes were eluted by using elution buffer (50 mM Tris–Cl, pH 7.4, 0.2% SDS, 0.1% Tween 20). Eluted proteins were subjected to western blot to confirm RdRp-host protein interaction using anti-FLAG antibody.

### RNA Immunoprecipitation and RT PCR

Huh7 S10-3 cells were harvested at ∼80% confluency in cold DPBS. Cells were lysed in lysis buffer (10 mM Tris–Cl, pH 7.4, 10 mM KCl, 2 mM MgCl_2_, 0.5% Tritin X-100 with protease inhibitor cocktail) for 20 min at 4°C followed by centrifugation at 12000 rpm at 4°C for 10 min. Clear supernatant was incubated with *in vitro* transcribed RNAs of either putative genomic or sub-genomic promoters of HEV for 2 h at 4°C on rotator. 25 μl of protein G dynabeads were incubated with 4 μg of anti-hnRNPK antibody produced in rabbit (Gene Tex) or anti-hnRNPA2B1 antibody produced in rabbit (Gene Tex) or a non-specific isotype IgG antibody in antibody binding buffer (PBS + 0.0.2% Tween 20) for 35 min at room temperature. Beads bound antibody complexes were added to previously incubated lysate plus RNA complex for 1 h at 4°C on rotator. Three washes of lysis buffer were given followed by a final wash of PBS. Complexes were eluted with 80 μl of elution buffer. Elutes were processed for total RNA isolation using phenol-chloroform method. The RNA was transcribed into cDNA using reverse transcriptase polymerase chain reaction (RT PCR) with Superscript III first strand synthesis kit followed by PCR amplification using Platinum taq DNA polymerase (Invitrogen) using specific primers for the detection of promoter regions. The PCR product was visualized on 2% Agarose gel.

## Results and Discussion

### Identification of HEV-Interacting Host Proteins

**FIGURE 1:**
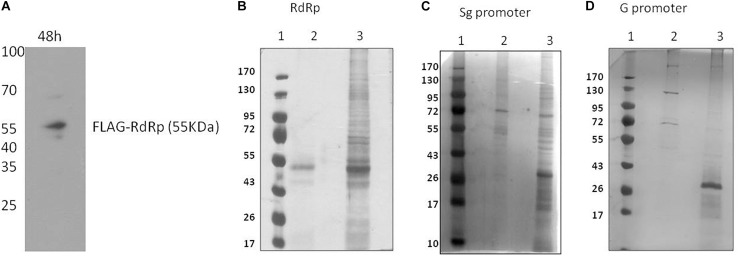
Identification of HEV interacting host proteins. **(A)** pcDNA_FLAG-RdRp construct was transfected into Huh7 S10-3 cells. Post 48 h of transfection cells were harvested and checked for expression of RdRp by western blot using anti-FLAG antibody. **(B)** Mock-transfected Huh 7 S10-3 cells or cells transfected with pcDNA_FLAG_RdRp (expressing FLAG-tagged RdRp) were harvested after 48 h of transfection. Immunoprecipetation was performed by anti-FLAG antibody or an isotype control antibody. RdRp interacting host proteins were eluted with protein G dynabeads and analyzed on SDS PAGE followed by silver staining. **(C)** HEV sub-genomic promoter (Sg) RNA interacting cellular proteins were pull down by using RNA affinity chromatography. Biotinylated HEV sub-genomic promoter RNA were immobilized on M280 streptavidin dynabeads. RNA immobilized beads were incubated with cell lysate of Huh7 S10-3. Interacting host proteins were eluted and checked on SDS PAGE followed by silver staining for visualization. Non-biotin RNA of the sub-genomic promoter was taken as control. **(D)** HEV genomic promoter (G) RNA interacting cellular proteins were pull down by using RNA affinity chromatography. HEV genomic promoter RNA interacting host proteins were eluted and checked on SDS PAGE followed by silver staining. For **(B–D)** lane 1, protein molecular weight ladder; lane 2, negative control pull-down; lane 3, experimental test pull down.

To pull down HEV RdRp-binding proteins, the HEV RdRp protein was expressed as a FLAG-tagged recombinant protein in Huh7 S10-3 cells. pcDNA_FLAG-RdRp construct was transfected in the cells, and the expression of RdRp was confirmed at 48 h post-transfection by western blotting using an anti-FLAG antibody (Figure 1A). Protein G magnetic beads were used to pull down specific interactions using the anti-FLAG antibody in a classical immunoprecipitation experiment from the RdRp-expressing cell lysate. The eluted complexes were subjected to 12% SDS-PAGE followed by silver staining with the ProteoSilver kit (Sigma) for visualizing protein bands (Figure 1B). Mock-transfected Huh7 S10-3 cell lysate was taken as control to detect the non-specific interactions. For the pull down of specific HEV RNA-interacting proteins, biotinylated HEV putative sub-genomic (Sg), or putative genomic promoter (G) RNA was synthesized *in vitro*. *In vitro* synthesized Sg RNA includes the recently mapped intragenomic promoter region regulating the Sg RNA transcription which is conserved across all HEV genotypes (Ding et al., 2018). Genomic promoter region has not been yet mapped functionally, however, based on the HEV RdRp binding studies reported previously with the 3′UTR of HEV anti-sense RNA, we have designed the putative G promoter RNA (Mahilkar et al., 2016). RNA affinity chromatography was performed using streptavidin magnetic beads with Huh7 S10-3 cell lysates incubated with HEV RNAs *in vitro*, and the elutes were subjected to protein identification. Non-biotin RNAs of sub-genomic and genomic promoters were taken as control. The eluted complexes were subjected to 12% SDS-PAGE followed by silver staining (Figures 1C,D). A distinct banding pattern could be observed in specific pull-down experiments as compared to the negative control. We further employed liquid chromatography quadrupole time-of-flight mass spectrometry (LC-QTOF/MS) to identify the interacting proteins. Proteins represented by at least two unique peptides and having less than 1% FDR were considered for analysis Proteins represented in respective negative control data were eliminated. The list of proteins identified to be interacting with HEV is shown in Table 2 and Supplementary Tables 1a–c.

**TABLE 2:** List of HEV genomic promoter, sub-genomic promoter, and RdRp interacting host proteins.

**HEV component**	**Gene symbol of interacting protein**
Sub-genomic promoter	HNRNPU, HNRNPA2B1, HNRNPF, NCL, CKAP4, HNRNPK, DDX3X, ACTA2, and POTEF
Genomic promoter	TUBA1B, TUBA1C, HSP90AA1, HSP90AB1, TUBB, PKM, CKB, FASN, ENO1, PRDX1, HSP90B1, PDIA6, RPLP2, HSPA5, PHGDH, KHSRP, PCBP2, CCT3, ALB, MYH9, VCP, SERPINH1, SPTBN1, ALDH1A1, VIM, SPTAN1, ATP5F1A, PRKDC, LMNA, HSPA9, PHB, SRSF1, DHX9, TUBB4B, HNRNPH1, HNRNPK, HNRNPA2B1, ACTN4, TUFM, HSPA6, HNRNPU, HIST1H1D, EEF1D, TPM3, H3F3B, HNRNPC, EEF2, RNH1, SFPQ, EEF1G, PCBP1, HNRNPAB, ATP5F1B, and HIST1H2BN
RNA dependent RNA polymerase	HNRNPA2B1, HNRNPK, DLST, HNRNPH1, OGDH, HIST3H2A, ACTBL2, EEF1A1, ACTB, and PFKL

*Proteins have been represented as NCBI gene names.*

### Construction and Analysis of HEV-Protein Interaction Network

**FIGURE 2:**
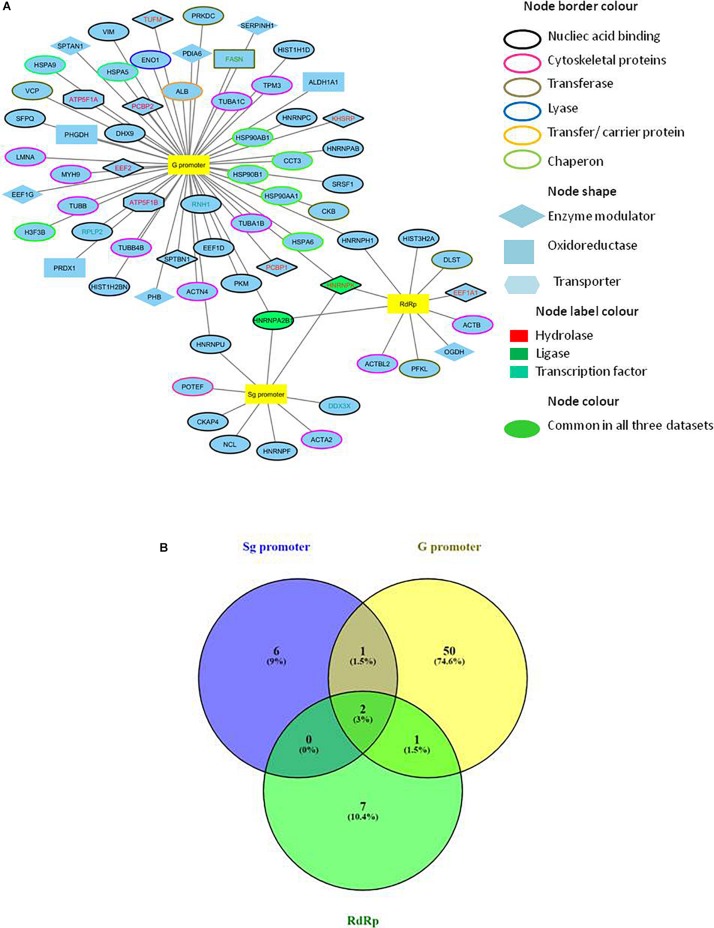
Construction of HEV-host interaction network. **(A)** HEV-host interaction network: Interaction map of HEV sub-genomic promoter (Sg), genomic promoter (G) and RdRp with interacting host proteins constructed in Cytoscape 3.6.1. Proteins were classified on the basis of their protein class by Panther gene ontology tool. The corresponding symbols indicating different protein classes have been mentioned on the figure. **(B)** Venn diagram comparing HEV interacting host proteins with different HEV components. Blue, yellow and green colors indicate proteins interacting with the sub-genomic promoter, genomic promoter, and RdRp, respectively. Common proteins within the data sets have been indicated in the colored intersections. Proteins have been represented as the respective NCBI gene names.

A combined list of proteins interacting with HEV promoters and RdRp was generated, and a protein network named the HEV-host interaction network was constructed using “Cytoscape version 3.6.1” (Figure 2A). A list of total 70 HEV-interacting proteins was generated, amongst which two proteins, HNRNPA2B1 and HNRNPK, were found to be present in all the three data sets. One more protein (HNRNPH) was found to be shared between RdRp and G promoter data, while another protein (HNRNPU) was common between the Sg promoter and G promoter besides HNRNPA2B1 and HNRNPK. However, about 94% of the proteins were found to be specifically interacting with only one of the HEV partners in the study (Figure 2B).

**FIGURE 3:**
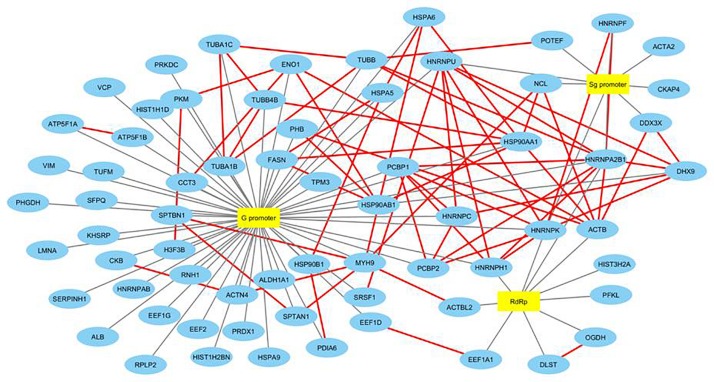
Construction and analysis of HEV-host PPI network. Interaction map of HEV interacting host proteins further interacting with the other proteins of our data. Black edges represent interactions revealed through mass spectrometry reported in this study. Secondary protein-protein interactions among host proteins revealed through literature mining have been indicated in red colored edges. Proteins have been represented as the respective NCBI gene names.

We then searched for the proteins interacting with other host proteins within our data to plot the protein-protein interaction network. We used advanced search options of IntAct database by providing “direct interactions” filter to find experimentally proven protein-protein interactions within our data and plotted a second network named HEV-host PPI network using Cytoscape 3.6.1 (Orchard et al., 2013). IntAct database reports evidences like pull down, X-ray crystallography, functional assays, etc under experimentally proven criteria of interaction prediction. IntAct tool sources the molecular interaction data from several curated databases like MINT, Uniprot, molecular connections, EMBL-EBI, and DIP. Primary interactions revealed through mass spectrometry in this study have been shown with black edges, while the secondary protein-protein interactions revealed through IntAct have been shown in red (Figure 3). We then calculated the topological parameters of the generated PPI network to access its modularity using the network analyzer tool of Cytoscape. It is revealed that 70 nodes representing HEV-host interactions were connected via a total of 141 edges. The average degree centrality, average path length distribution, and the clustering coefficient of the network were observed to be 0.748, 2.365, and 0.348, respectively. The topological parameters were compared with HEV-human protein interaction networks previously reported in literature which validated the significance of HEV-host protein interaction network constructed in our study (Supplementary Table 2). Amongst the proteins present in our data, PFKL, SERPIN, HNRNPH, HSP90AB1, and TUBB have been previously reported to interact with HEV macrodomain (Ojha and Lole, 2016). Besides, PCBP1 and EEF1A1 have been reported to interact with HEV ORF2, while DHX9, HNRNPC, and HNRNPK have been reported to interact with HEV non-coding regions on the sense strand (Paingankar and Arankalle, 2015; Subramani et al., 2018). This confirms the authenticity of HEV-specific interactome found in our study.

**FIGURE 4:**
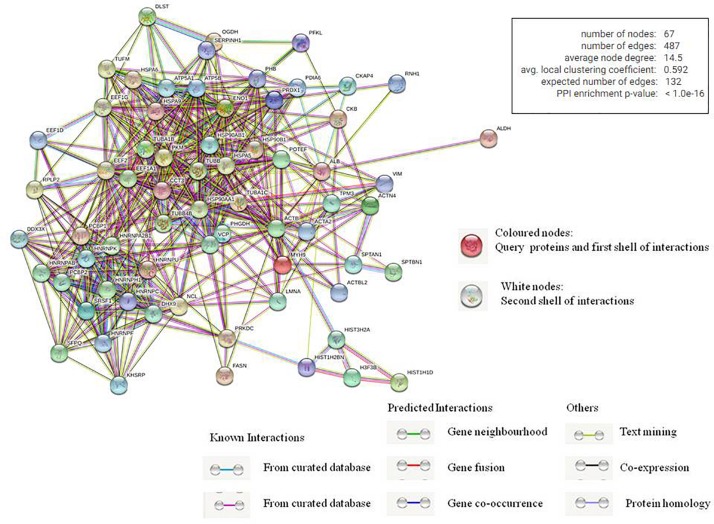
Analysis of inter protein interaction network using STRING database. Each edge color indicates a different method of protein-protein interaction prediction as indicated below the figure.

We also used STRING database to generate the inter-protein interaction network from our data (Figure 4 and Supplementary Table 3) (Szklarczyk et al., 2015). This analysis gave us a more intense multidimensional network as, along with the experimentally determined interactions, those predicted from gene fusion; co-expression, homology, and text mining were also considered. The observed number of edges for the network (487) was significantly higher than the expected number of edges (132) for the given number of nodes (67), implying that there are more interactions than expected. The result suggested that the proteins in our data dispaly more interactions than expected for a random set of proteins. Such enrichment indicates that the proteins are at least partially biologically connected as a group, highlighting the significance of the HEV-host network reported in this study. This also indicates the probability of isolating HEV-specific protein complexes gathered at the HEV replication site.

### Gene Ontology Annotation

To investigate the cellular components or pathways that have been enriched in the HEV-host protein interaction network, we performed gene ontology analysis (GO annotation) of the data using various web-based tools. The GO annotation resulted in classification of the proteins based on their functional clusters or GO categories. The enrichment analysis performed using Panther (Gene Ontology Consortium’s web tool) (Supplementary Figures 1A–C, 2), Gprofiler (Supplementary Figure 3), STRING (Supplementary Figure 4), and Enricher was in agreement and showed a significant enrichment of similar GO terms. FDR <0.05 was set as a statistical threshold for GO analysis. Enrichment of proteins in three different categories, namely biological processes, molecular function, and cellular component, was performed using Enricher (Chen et al., 2013; Kuleshov et al., 2016). Proteins were arranged according to the combined enrichment score, which is a combination of the *p*-value and z-score calculated by multiplying the two scores. The *p*-value is the probability of any gene belonging to any set and is calculated by using exact Fisher’s test. The Z-score is calculated by using a modified Fisher’s exact test and assesses the deviation from the expected rank. The combined score provides a compromise between the available methods for multiple test corrections to control the FDRs (Kuleshov et al., 2016).

**FIGURE 5:**
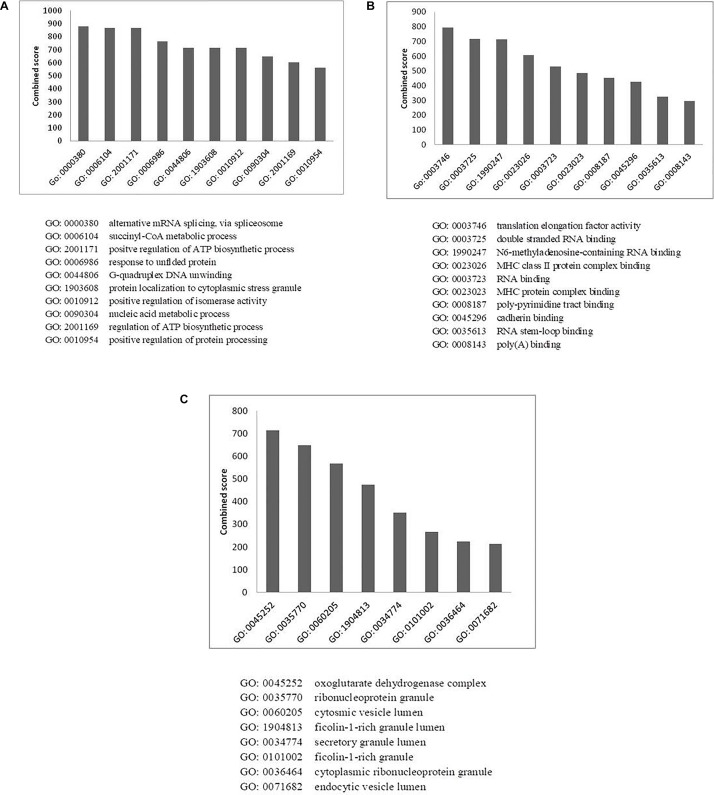
Gene ontology analysis of HEV- host interactions based on **(A)** biological process, **(B)** molecular function, and **(C)** cellular component category. *Y*-axis represents the combined enrichment score computed using Enricher.

Enrichment of GO terms in biological process category revealed the enrichment of proteins related to mRNA splicing (GO: 0000380; *p*-value: 3.5 × 10^–3^), response to unfolded proteins (GO: 0006986; *p*-value: 3.6 × 10^–6^), nucleic acid metabolic process (GO: 0090304, *p*-value: 1.3 × 10^–6^), and regulation of protein processing (GO: 0010954, *p*-value: 8 × 10^–3^) (Figure 5A and Supplementary Table 4A). A vital characteristic of any virus-host interaction is the manipulation of cellular gene expression in order to establish favorable cellular conditions for efficient viral replication. Eukaryotic genes are highly regulated at post-transcriptional stages, such as splicing, export, regulation of translation, subcellular localization and mRNA turnover. Viruses have often been observed to target these RNA processing stages to hijack the host RNA metabolism which is evident from the enrichment of host RNA splicing and nucleic acid metabolism related protein in our data. Such enhancement could also hint at the reliance of HEV on host systems for the regulation of viral RNA translation, transcription, and stability within the host cell (Ahlquist et al., 2003). We observed the highest number of proteins having nucleic acid-binding activity in our data, which further confirms the dependence of HEV on the host for viral RNA metabolism. We have incorporated the data of classification of proteins on the basis of protein class in the HEV-host interaction network using different color codes as indicated on the figure (Figure 2A and Supplementary Figure 2).

We also observed enrichment of stress granule proteins (GO:1903608; *p*-value: 3.3 × 10^–2^) in our data. Stress granules are dynamic structures that are formed when cellular translational rates decline after external stresses are applied to cells. Viral infection into the host cell has been shown to induce cytoplasmic stress granule formation in the host cells due to the upregulation of stress granule proteins, such as helicase DDX3X and DHX9 (Ariumi et al., 2011; Pène et al., 2015). As stress granules regulate the cycle of mRNA turnover and gene expression, they happen to be another vital point for viruses to manipulate cellular systems. Stress granules have also been reported to play a role in promoting innate immune responses (Reineke and Lloyd, 2013). This is an additional reason why viruses must counteract the effects of such granules for efficient replication by interacting with proteins of stress granules.

Virus replication alters the normal metabolic processes of the host by interacting with components of different molecular pathways. Enrichment of GO terms in molecular function category also resulted in enrichment of proteins involved in RNA metabolism processes, which again highlights the need of the virus to interact with proteins of host RNA turnover. The enriched proteins in molecular function category belong to Translation elongation factor activity (GO: 0003746, *p*-value: 1 × 10^–3^), RNA binding (GO: 0003723, *p*-value: 3.9 × 10^–25^) and RNA stem loop binding (GO: 0035613, *p*-value: 3.4 × 10^–2^).

MHC protein complex-binding proteins (GO: 0023023, *p*-value: 2.5 × 10^–3^) have also been observed to be enriched in the molecular function category (Figure 5B and Supplementary Table 4B). The anti-viral responses of the host and the invading strategies of the pathogens have evolved concurrently for millions of years. Infecting pathogens have developed several escape strategies to cripple the immune system. Several viruses have evolved with proteins that interfere with antigen presentation and which target both MHC-I and MHC-II antigen processing pathways in order to distort the anti-viral immune response of the host. Several viruses such as HSV, Epstein-Barr virus, Bovine herpes virus and cytomegalovirus have been shown to have evolved strategies to combat MHC-mediated host immunity (Yewdell and Bennink, 1999; Røder et al., 2008). However, there are no literature reports of HEV interactions with MHC molecules. In the HEV interactome reported in this study, we observed the presence of MHC-interacting proteins, such as HSP90AA1 and PKM, which could be explored further for their ability to alter host immune response in the context of HEV infection.

Enrichment analysis of GO terms according to cellular component enhanced the representation of GO terms, such as ribonucleoprotein granules (GO: 0035770, *p*-value: 4 × 10^–8^), secretory granule lumen (GO: 0034774, *p*-value: 1 × 10^–9^) and endocytic vesicle lumen (GO: 0071682, *p*-value: 3.8 × 10^–2^) (Figure 5C and Supplementary Table 4C). HEV has been reported to enter liver cells through receptor-mediated endocytosis (Kapur et al., 2012). In agreement with this, our data also suggest enrichment of proteins belonging to endocytic vesicle lumen. HEV replication takes place in the cytoplasm. Along with the replicase complex, HEV RdRp has been shown to localize to the endoplasmic reticulum (ER) membrane (Rehman et al., 2008). A recent report also concluded that ORF1 polyprotein co-localizes with the markers of ER-Golgi intermediate compartment, suggesting the involvement of secretory pathway during replication (Szkolnicka et al., 2019). Previous studies have also indicated that HEV forms membrane-associated particles in the cytoplasm by means of budding into intracellular vesicles. HEV exploits the multivesicular body pathway to release infectious virion particles outside the cell through the cellular exosomal pathway (Nagashima et al., 2014).

### Pathway Enrichment Analysis

**FIGURE 6:**
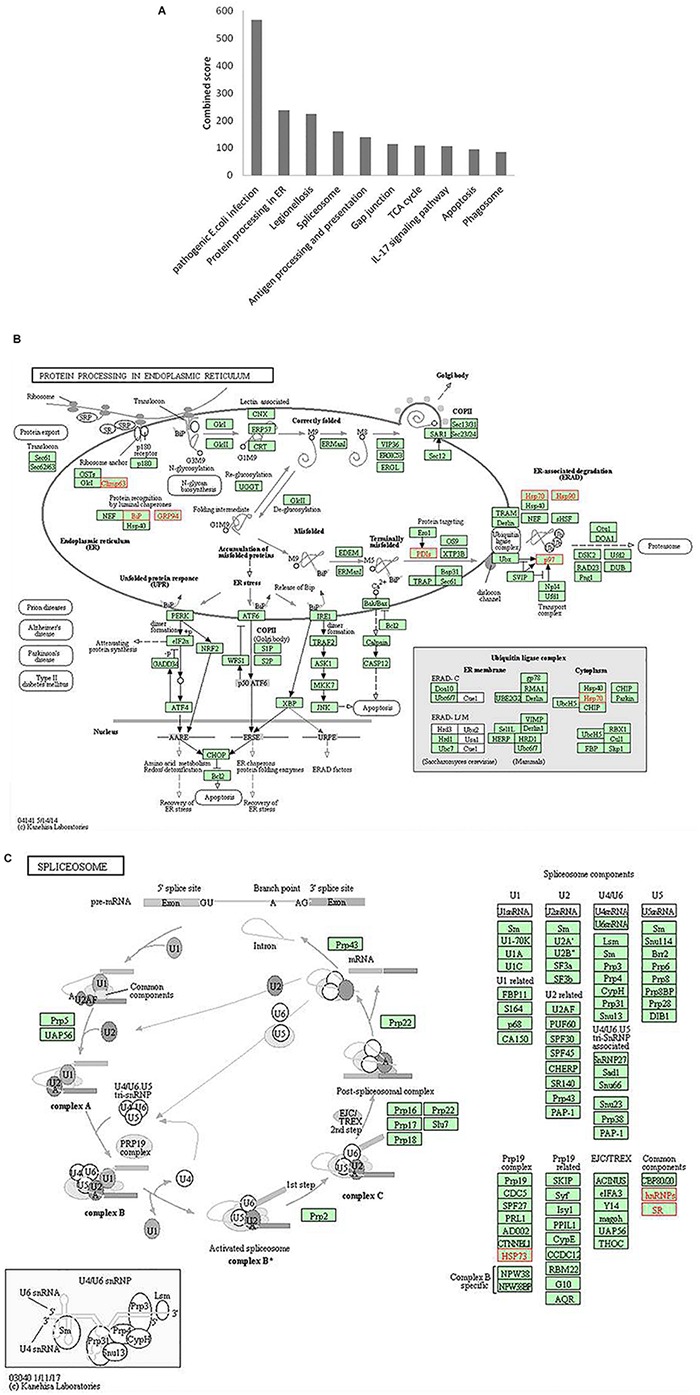
Pathway enrichment analysis. **(A)** Graph shows the enriched pathways targeted by HEV, analyzed by KEGG functional annotation pathway database. *Y*-axis represents the combined score computed using Enricher. **(B)** Schematic representation of “protein processing in endoplasmic reticulum” pathway (imported from KEGG: map04141). **(C)** Schematic representation of the spliceosome pathway targeted by HEV host interacting proteins (imported from KEGG: map03040). Proteins interacting with HEV RNA promoter (G or Sg) or polymerase within the entire pathway are shown in red color.

To understand the cellular pathways targeted by HEV, we performed pathway enrichment analysis by using the KEGG functional annotation pathway database through Enricher. The results revealed enrichment of different pathways involving pathogenic *Escherichia coli* infection, protein processing in endoplasmic reticulum, legionellosis, spliceosome, antigen presentation and processing, gap junction, citrate cycle, IL17 signaling pathways, apoptosis, and phagosome (Figure 6A and Supplementary Table 5). Furthermore, the schematic of the entire pathway network for endoplasmic reticulum protein processing and spliceosome pathway was obtained from the KEGG pathway database (Kanehisa and Goto, 2000; Kanehisa et al., 2019).

Protein processing in the endoplasmic reticulum pathway was enriched (*p*-value 1.2 × 10^–5^). Seven proteins of this pathway, namely, CKAP4, HSPA5, HSP90B1, PDIs, HSP70, HSP90, and VCP were found to interact with HEV RNA and polymerase (Figure 6B). These factors are involved in proper folding and processing of newly synthesized proteins in the endoplasmic reticulum. As HEV virus replicates on the ER membrane and its polymerase localizes onto the ER, its interaction with proteins residing at the endoplasmic reticulum is apparent during its replication.

Proteins belonging to the heat shock protein (HSP) family are well known for their roles as chaperons in protein folding. These proteins have been observed to play similar roles in the maintenance of proper viral protein folding and stabilization (Seo et al., 2018). In many viral infections, viruses commandeer vital cellular components, leading to cellular stress. Cellular stress is often represented as unfolded protein response (UPR) in cells (Geller et al., 2012; Wang et al., 2018). Previous studies have reported that ORF3 and ORF2 of the virus induce UPR and ER stress (Surjit et al., 2007; John et al., 2011; Xu et al., 2014). In our study, we found enrichment of proteins related to processing in the endoplasmic reticulum, and UPR further confirms the dependence of HEV on the host ER machinery.

In our analyses, the spliceosome pathway was found to be enriched by *p*-value of 5.9 × 10^–4^). Different heterogeneous nuclear ribonucleoproteins, such as HNRNPK, HNRNPA2B1, HNRNPH, HNRNPE1, HNRNPE2, HNRNPC and HNRNPU, and other spliceosomal accessory complex proteins, such as HSP73 and SR, interact with HEV (Figure 6C). Spliceosomal complex proteins help in the generation of stable RNA structures, while ribonucleoproteins play roles in RNA stability and transport (Will and Lührmann, 2011). Previous studies have reported the binding of HNRNPA2B1 to Influenza virus and Dengue virus RNA and explained its role in regulation of viral transcription (Paranjape and Harris, 2007; Wang et al., 2014). HNRNPK binds to the core protein of Dengue virus, whereas in Sindbis virus infection, it binds to its non-structural protein and sub-genomic RNA to regulate viral replication (Chang et al., 2001; Burnham et al., 2007; LaPointe et al., 2018). HNRNPK binds to hepatitis C virus RNA near the miR-122 binding site to facilitate its replication (Fan et al., 2015). In another study, the role played by HNRNPK in HCV virion production is reported to be mediated by viral RNA binding (Poenisch et al., 2015). HNRNPK binds to Influenza M1 RNA and regulates its splicing while maintaining appropriate ratio of M2/M1 protein (Thompson et al., 2018). HNRNPH binds to the negative regulator of splicing elements in Rous sarcoma virus to regulate the splicing and polyadenylation machinery (Fogel and McNally, 2000). A quantitative proteomics study by Rogée et al. (2015) evaluating the alteration of host factors during HEV infection in swine revealed an upregulation of HNRNPK in infected livers (Rogée et al., 2015). In another study of modulation of host factors during HEV infection in A549 cells, a significant increase in the expression of HNRNPH was observed in response to virus infection (Shen et al., 2014). Our study is consistent with these findings as it underlines a putative role of hnRNP proteins in modulating HEV RNA transcription by binding at the promoter site and HEV polymerase.

### Validation of Interactions Between HNRNPK and HNRNPA2B1 With HEV Promoters and RdRp

**FIGURE 7:**
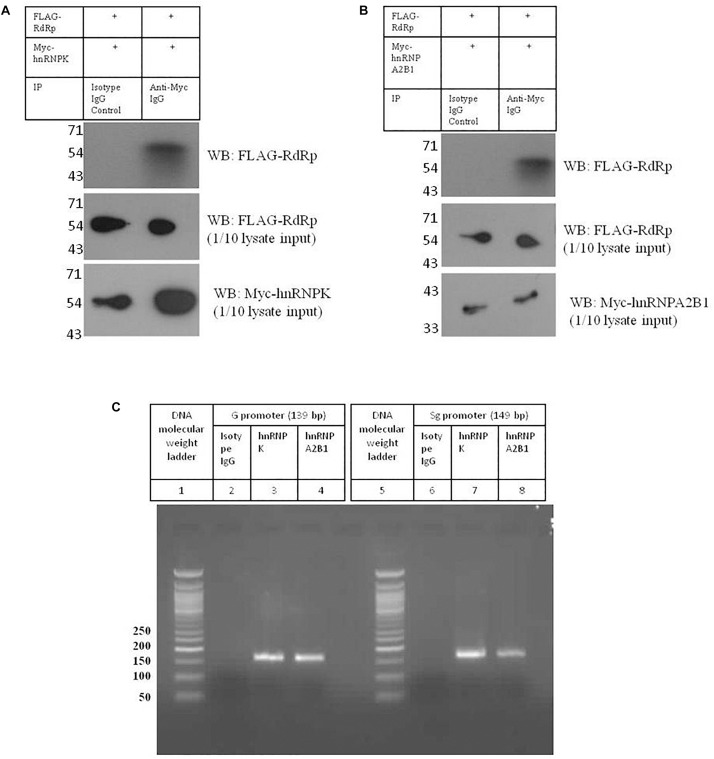
Validation of interactions between HNRNPK and HNRNPA2B1 with HEV promoters and RdRp. **(A)** pTandem_FLAG-RdRp_Myc-HNRNPK plasmid was transfected in Huh 7 S10-3 cells. 48 h post transfection co-IP was performed with anti c-Myc antibody. Interaction of c-Myc tagged HNRNPK with that of FLAG tagged RdRp was checked with western blot by using anti c-Myc antibody. **(B)** pTandem_FLAG-RdRp_Myc-HNRNPA2B1 plasmid was transfected in Huh 7 S10-3 cells. 48 h post transfection co-IP was performed with anti c-Myc antibody. Interaction of c-Myc tagged HNRNPA2B1 with that of FLAG tagged RdRp was checked with western blot by using anti c-Myc antibody. **(C)** Huh 7 S10-3 cell lysate was incubated with HEV G and Sg promoter RNA followed by immunoprecipitation with anti HNRNPK or anti HNRNPA2B1 antibody. RT-PCR was performed to detect HNRNP-bound HEV RNAs in the elutes. Figure shows amplified PCR products on 2% agarose gel.

Analysis of HEV-host protein interactions suggested that HNRNPK and HNRNPA2B1 are common factors associating with HEV RNA promoters and polymerase (Figures 2A,B). To demonstrate the validity of the interactions obtained through mass spectrometry, we further *in vitro* validated the interactions between HNRNPK and HNRNPA2B1 with that of HEV promoters and RdRp. In order to confirm the interaction of RdRp with host proteins, coimmunoprecipitation technique was employed. HEV RdRp encoding sequence and the coding sequence of selected host protein were cloned in pTandem vector. pTandem vector was chosen for its additional feature which enables cloning of two different genes in the same construct to increase the co-transfection efficiency. Huh7 S10-3 cells transfected with pTandem_Flag-RdRp_Myc-HNRNPK or pTandem_Flag-RdRp_Myc-HNRNPA2B1 plasmids. Post 48 h of transfection co-immunoprecipitation was performed by using anti myc antibody or isotype IgG antibody. After co-immunoprecipitation, interaction of host factors with RdRp was confirmed by western blot using anti FLAG antibody. We observed that both HNRNPK and HNRPA2B1 specific immunoprecipitation could pull down HEV RdRp (Figures 7A,B). Our results confirmed the binding of both of these proteins with HEV RdRp as obtained in the mass spectrometry data.

To further confirm the binding of HNRNPK and HNRNPA2B1 with HEV putative promoters; we employed RNA immunoprecipitation followed by RT PCR. Huh7 S10-3 cell lysate was incubated with G or Sg promoter RNA followed by pull down by anti-HNRNPK or anti-HNRNPA2B1 or isotype IgG antibody. From the eluted complexes, total RNA was isolated and presence of promoter region RNA sequence was checked by RT PCR. We observed that both HNRNPK and HNRNPA2B1 could successfully pull down HEV genomic and sub-genomic promoter RNA as evident by the specific band on agarose gel (Figure 7C). Therefore we could successfully demonstrate the *in vitro* binding of HNRNPK and HNRNPA2B1 with HEV RdRp and HEV promoters as obtained from the mass spectrometry data, further increasing the confidence level of the obtained interactions. Thus, we believe that HNRNPK and HNRNPA2B1 play crucial roles in HEV replication and their functional significance in the context of HEV replication can be further assessed. Taken as a whole, our study reveals the importance of host cellular machinery in HEV lifecycle regulation. Studying host pathways targeted by HEV can facilitate the hunt for putative anti-viral candidates for therapeutic purposes. In conclusion, our study shows that analyzing host-virus interactions through system biology approach can be beneficial in understanding the molecular regulation of viral lifecycle and can put forth set testable hypotheses for future experimental validation.

## Data Availability Statement

All datasets generated/analyzed for this study are included in the article/Supplementary  Material.

## Author Contributions

GK, KP, and YK conceived and designed the experiments, analyzed the data, and wrote the manuscript. GK and KP performed the experiments.

## Conflict of Interest

The authors declare that the research was conducted in the absence of any commercial or financial relationships that could be construed as a potential conflict of interest.
