# Supplementary material for: Protein Interactions Network of Hepatitis E Virus RNA and Polymerase With Host Proteins
Source: Front Microbiol. 2019 Nov 1;10:2501. doi: 10.3389/fmicb.2019.02501 (PMC6838024; doi:10.3389/fmicb.2019.02501)
Supplement: Supplementary file 1 [file Data_Sheet_1.zip › Data Sheet.docx]

**Supplementary figures and tables:**

**Supplementary Fig. 1a.**


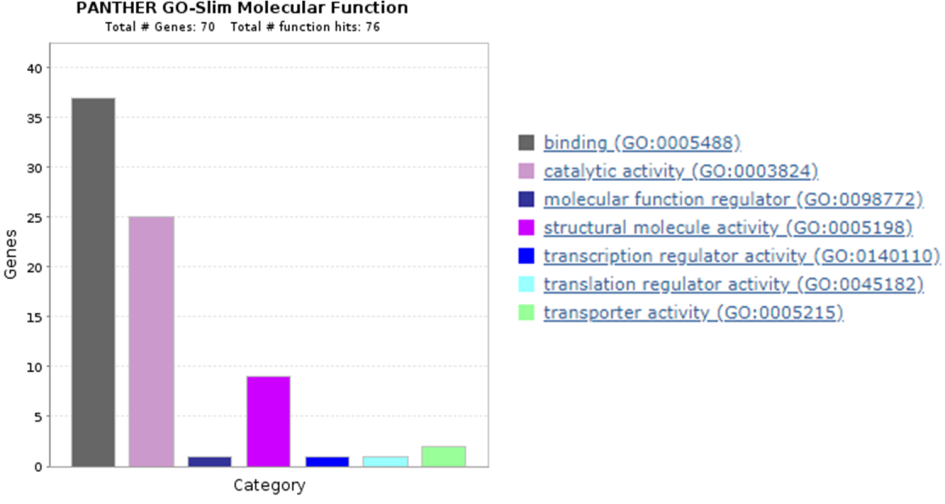


**Supplementary Fig. 1b**


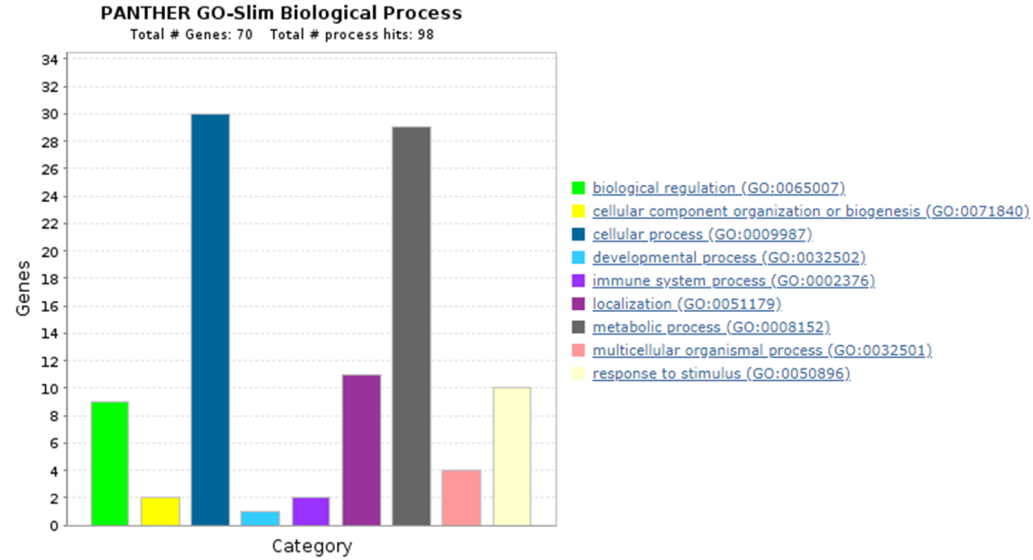


**Supplementary Fig. 1c**


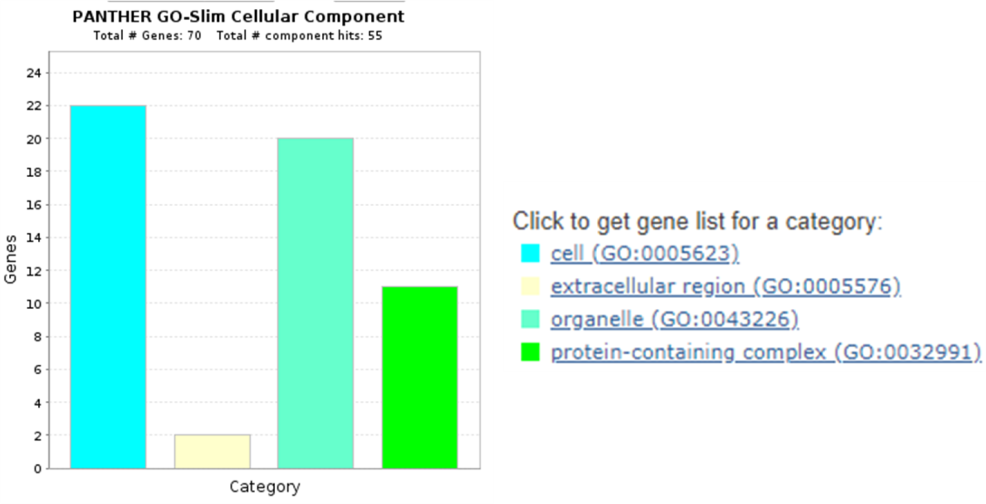


**Supplementary Fig. 1:** Gene set enrichment analysis performed using Panther (Gene Ontology Consortium’s web tool). a) Molecular function, b) Biological process & c) Cellular component.

**Supplementary Fig. 2**


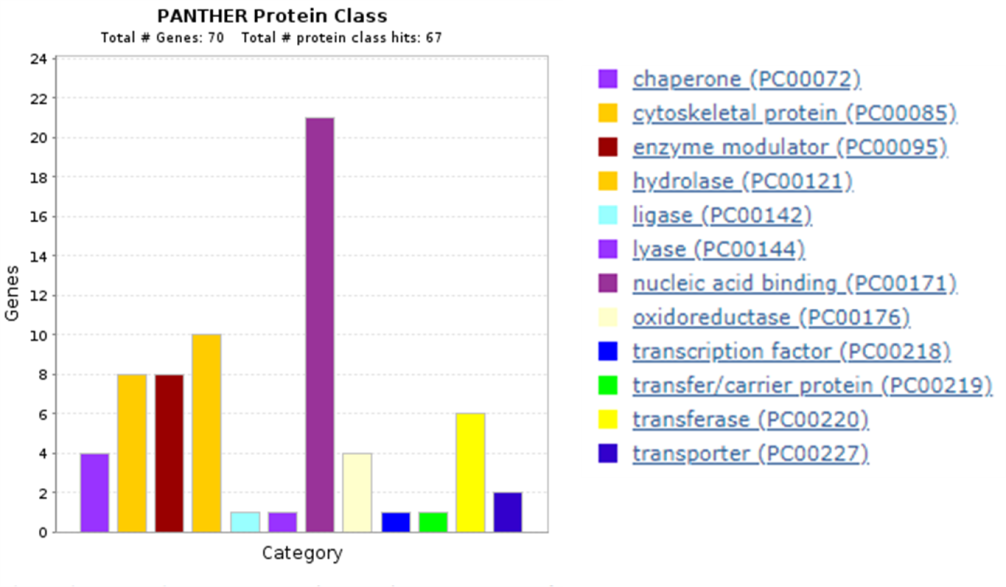


**Supplementary Fig. 2:** Classification of HEV interacting host proteins by using Panther based on protein class (Gene Ontology Consortium’s web tool).

**Supplementary Fig.3**


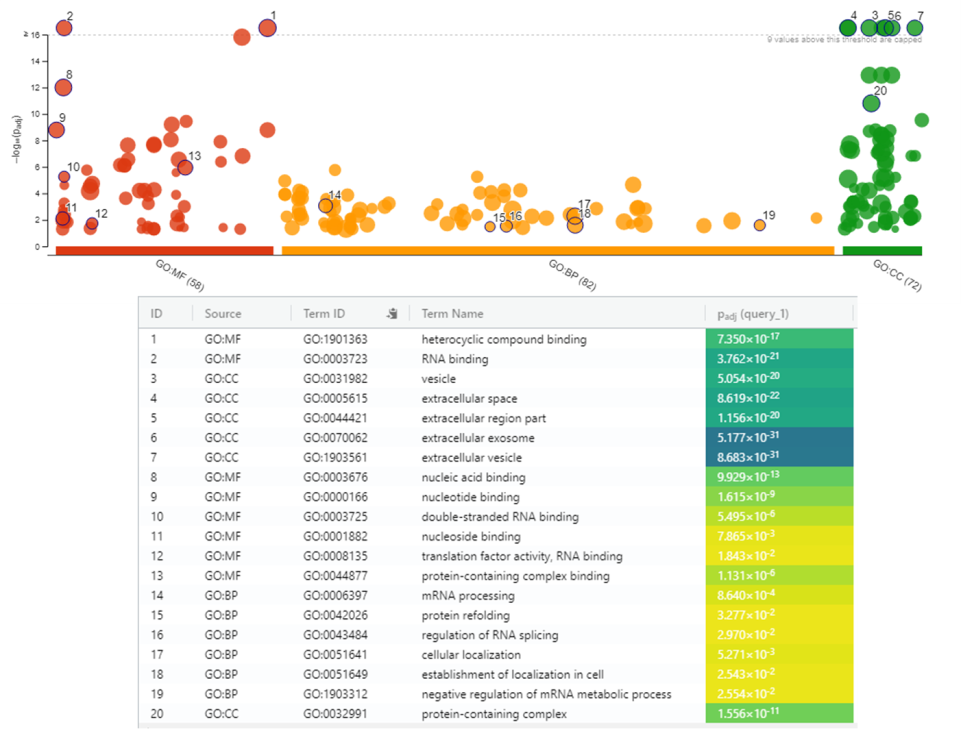


**Supplementary fig. 3:** Gene set enrichment analysis performed using Gprofiler. Red, orange and green indicate proteins belonging to molecular function, biological process and cellular component categories. Numbered dots have been enlisted in the table below representing the name of respective enriched GO category.

**Supplementary Fig.4**


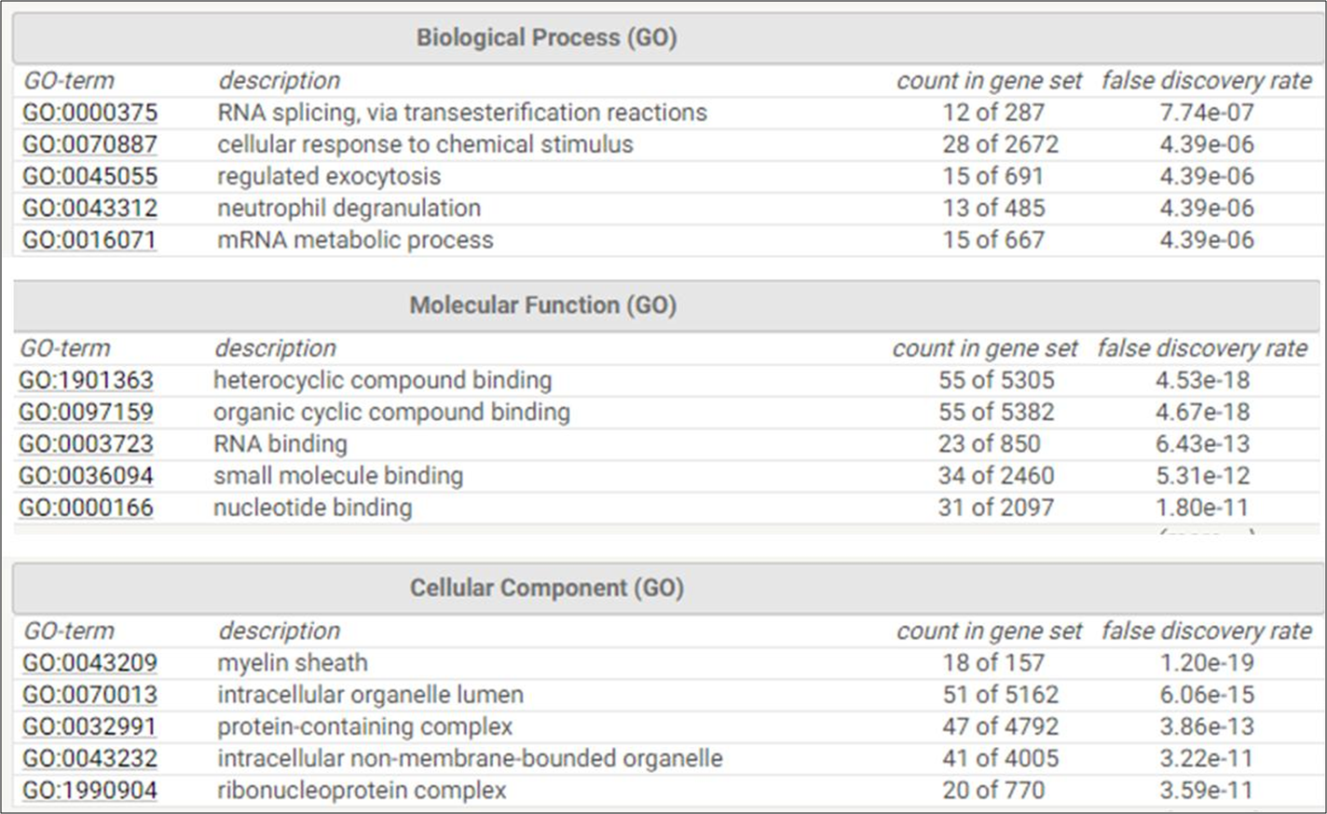


**Supplementary Fig.4:** Gene set enrichment analysis in molecular function, biological process and cellular component categories performed using STRING.

**Supplementary table 1:**

**Supplementary table 1a:**

| **Uniprot ID** | **Protein name** | **Gene name** | **Functional catagory** |
| --- | --- | --- | --- |
| P22626 | Heterogeneous nuclear ribonucleoprotein A2/B1 | HNRNPA2B1 | Spliceosome component |
| P61978-2 | Heterogeneous nuclear ribonucleoprotein K | HNRNPK | Central molecule of spliceosome |
| P36957 | Dihydrolipoyllysine-residue succinyltransferase | DLST | Acetyl coA catabolism |
| G8JLB6 | Heterogeneous nuclear ribonucleoprotein H | HNRNPH1 | Spliceosome component |
| E9PCR7 | oxoglutarate dehydrogenase, mitochondrial | OGDH | tricarboxylic acid cycle |
| Q7L7L0 | Histone H2A type 3 | HIST3H2A | DNA repair |
| Q562R1 | Beta-actin-like protein | ACTBL2 | Cellular cytoskeleton associated |
| P68104 | Elongation factor 1-alpha | EEF1A1 | Translation elongation |
| P60709 | Actin | ACTB | Cytoskelton |
| P17858-2 | ATP-dependent 6-phosphofructokinase, liver type | PFKL | Glucose metabolism |

**Supplementary table 1b:**

| **Uniprot ID** | **Protein name** | **Gene name** | **Functional category** |
| --- | --- | --- | --- |
| Q00839 | Heterogeneous nuclear ribonucleoprotein U | HNRNPU | Spliceosome component |
| P22626 | Heterogeneous nuclear ribonucleoprotein A2/B1 | HNRNPA2B1 | Spliceosome component |
| P52597 | Heterogeneous nuclear ribonucleoprotein F | HNRNPF | Spliceosome component |
| P19338 | Nucleolin | NCL | Chromatin decondensation |
| Q07065 | Cytoskeleton ssociated protein 4 | CKAP4 | Cell surface receptor |
| P61978-2 | Heterogeneous nuclear ribonucleoprotein K | HNRNPK | Central molecule of spliceosome |
| O00571 | ATP-dependent RNA helicase | DDX3X | ATP dependent RNA helicase activity |
| P62736 | Actin | ACTA2 | Cytoskelton |
| A5A3E0 | POTE ankyrin domain family member | POTEF | Blood microparticle |

**Supplementary table 1c:**

| **Uniprot ID** | **Protein name** | **Gene name** | **Function of the protein** |
| --- | --- | --- | --- |
| P68363 | Tubulin alpha-1B | TUBA1B | Cytoskeleton-dependent intracellular transport, GTPase activity |
| F5H5D3 | Tubulin alpha-1C | TUBA1C | Structural constituent of cytoskeleton, GTPase activity |
| P07900 | Heat shock protein HSP 90-alpha | HSP90AA1 | Molecular chaperone |
| P08238 | Heat shock protein HSP 90-beta | HSP90AB1 | Molecular chaperone |
| P07437 | Tubulin beta chain | TUBB | Cytoskeleton-dependent intracellular transport, GTPase activity |
| P14618 | Pyruvate kinase | PKM | Puruvate biosynthesis |
| P12277 | Creatine kinase B | CKB | Creatine metabolic process |
| P49327 | Fatty acid synthase | FASN | Fatty acid metabolism |
| P06733 | Alpha-enolase | ENO1 | Glucose metabolism |
| Q06830 | Peroxiredoxin-1 | PRDX1 | Cellular response to oxidative stress |
| P14625 | Endoplasmin | HSP90B1 | Molecular chaperone |
| Q15084 | Protein disulfide-isomerase A6 | PDIA6 | Endoplasmic reticulum chaperon |
| P05387 | 60S acidic ribosomal protein P2 | RPLP2 | mRNA catabolism |
| P11021 | 78 kDa glucose-regulated protein | HSPA5 | Endoplasmic reticulum chaperon |
| O43175 | D-3-phosphoglycerate dehydrogenase | PHGDH | Amino acid metabolism |
| Q92945 | Far upstream element-binding protein 2 | KHSRP | mRNA processing and transport |
| Q15366 | Poly(rC)-binding protein 2 | PCBP2 | mRNA metabolism, Innate antiviral immune response |
| P49368 | T-complex protein 1 subunit gamma | CCT3 | Chaperon for protein folding |
| P02768 | Serum albumin | ALB | Blood microparticle |
| P35579 | Myosin-9 | MYH9 | Actin cytoskeleton mediated cell migration |
| P55072 | Transitional endoplasmic reticulum ATPase | VCP | Endoplasmic reticulum formation |
| P50454 | Serpin H1 | SERPINH1 | Collagen binding |
| A0A087WUZ3 | Spectrin beta | SPTBN1 | Actin binding |
| P00352 | Retinal dehydrogenase 1 | ALDH1A1 | Cellular aldehyde metabolic process |
| P08670 | Vimentin | VIM | Structural constituent of cytoskeleton |
| A0A0D9SGF6 | Spectrin alpha | SPTAN1 | Microtubule cytoskeleton |
| P25705 | ATP synthase subunit alpha, mitochondrial | ATP5F1A | Mitochondrial proton-transporting ATP synthase complex |
| P78527 | DNA-dependent protein kinase catalytic subunit | PRKDC | DNA repair pathway |
| P02545 | Prelamin-A/C | LMNA | Component of nuclear lamina |
| P38646 | Stress-70 protein, mitochondrial | HSPA9 | Mitochondrial chaperon |
| P35232 | Prohibitin | PHB | Regulation of cell proliferation |
| J3KTL2 | Serine/arginine-rich-splicing factor 1 | SRSF1 | RNA binding |
| Q08211 | ATP-dependent RNA helicase A | DHX9 | ATP-dependent 3'-5' DNA/RNA helicase activity |
| P68371 | Tubulin beta-4B | TUBB4B | Structural constituent of cytoskeleton |
| G8JLB6 | Heterogeneous nuclear ribonucleoprotein H | HNRNPH1 | RNA binding |
| P61978-2 | Heterogeneous nuclear ribonucleoprotein K | HNRNPK | Central molecule of spliceosome |
| P22626 | Heterogeneous nuclear ribonucleoprotein A2/B1 | HNRNPA2B1 | Spliceosome complex component |
| O43707 | Alpha-actinin-4 | ACTN4 | Cytoskeleton |
| P49411 | Elongation factor Tu | TUFM | Translation elongation |
| P17066 | Heat shock 70 kDa protein 6 | HSPA6 | Chaperon for protein folding |
| Q00839 | Heterogeneous nuclear ribonucleoprotein U | HNRNPU | Spliceosome complex component |
| P16402 | Histone H1.3 | HIST1H1D | Regulation of chromatin silencing |
| H0YCK7 | Elongation factor 1-delta | EEF1D | Translation elongation |
| P06753 | Tropomyosin alpha-3 | TPM3 | Structural constituent of cytoskeleton |
| K7ES00 | Histone H3.3 | H3F3B | Protein heterodimerization |
| P07910 | Heterogeneous nuclear ribonucleoprotein C1/C2 | HNRNPC | Spliceosome complex component |
| P13639 | Elongation factor 2 | EEF2 | Translation elongation |
| P13489 | Ribonuclease inhibitor | RNH1 | mRNA catabolism |
| P23246 | Splicing factor, proline- and glutamine-rich | SFPQ | Transcription regulation |
| P26641 | Elongation factor 1-gamma | EEF1G | Translation elongation |
| Q15365 | Poly(rC)-binding protein 1 | PCBP1 | RNA metabolism |
| D6RBZ0 | Heterogeneous nuclear ribonucleoprotein A/B | HNRNPAB | RNA binding |
| P06576 | ATP synthase subunit beta, mitochondrial | ATP5F1B | Mitochondrial proton-transporting ATP synthase complex |
| U3KQK0 | Histone H2B | HIST1H2BN | Protein heterodimerization |

**Supplementary table 1:** List of HEV host interacting proteins along with their Uniprot ID, protein name, gene name and function. a) HEV RdRp interacting host proteins, b) HEV genomic promoter interacting host proteins and c) HEV sub-genomic promoter interacting host proteins.

**Supplementary table 2:**

| Name of the topological parameter of the network | HEV-host PPI network from this study | HEV-human PPIN (Subramani et al., 2018) | HEV H-H-ORF1 network (Ojha &Lole., 2015) |
| --- | --- | --- | --- |
| Clustering coefficient | 0.348 | 0.021 | 0.059 |
| Centralization | 0.748 | 0.279 | 0.037 |
| Characteristic path length | 2.365 | 3.6 | 4.438 |
| No of nodes | 70 | 148 | 155 |
| No of edges | 141 | 188 | 184 |

**Supplementary table 2:** Comparison of topological parameters of HEV interaction networks

**Supplementary table 3: (**has been provided as a separate excel file): Protein-protein interactions data obtained from STRING. The methods of interaction prediction have been listed.

**Supplementary table 4a:**

**Supplementary table 4b:**

**Supplementary table 4c:**

**Supplementary table 4:** List of enriched GO terms using Enrichr along with corresponding genes involved by a) biological process category b) molecular function category and c) cellular component category.

**Supplementary table 5:**

**Supplementary table 5:** List of enriched pathways predicted using KEGG database along with corresponding genes involved.
